# Antimicrobial resistance surveillance with whole genome sequencing in Africa: It’s (about) time

**DOI:** 10.4102/ajlm.v7i2.761

**Published:** 2018-12-06

**Authors:** Hajo Grundmann, Hellen Gelband

**Affiliations:** 1 Institute for Infection Prevention and Hospital Epidemiology, Medical Centre, University of Freiburg, Freiburg, Germany; 2 Centre for Global Health Research, University of Toronto, Ontario, Canada; 3 Global Public Health Consulting, Takoma Park, Maryland, United States

## Introduction

The current move to establish a global system for antimicrobial resistance (AMR) surveillance was born of the need for reliable data that would allow estimating the impact of AMR on public health and the economy, strengthen the evidence for acting, and point toward appropriate interventions. Surveillance represents an important component of the global action plan on AMR adopted by the 68th World Health Assembly and embodied in the World Health Organization’s developing Global Antimicrobial Resistance Surveillance System (GLASS).^1^ GLASS is partly predicated on reproducing programmes that have been successful in Europe and elsewhere, following a longstanding and often successful tradition of transferring public health strategies from richer to poorer countries. The main aim is to generate datasets for trend analysis and benchmarking across nations. This is an important and useful purpose, mainly to guide policy decisions and allocate resources where and when predictable threats to public health need to be addressed. At the same time, it should not be seen as a reason to delay the introduction of another layer that is already being added in Europe^2,3^ - whole genome sequencing (WGS) enrich our understanding of AMR evolution and spread and contribute practical information for local and national infection control and clinical guidance. Our view is that WGS-based surveillance could and should be developed now in Africa in parallel, and may actually produce informative results more quickly until the conventional approach renders authoritative and comparable data. Our rationale is explained in this article.

## Background: Prospects for laboratory-based surveillance in Africa and novel opportunities

The most accomplished example of repurposing routine laboratory susceptibility results for AMR surveillance and the basic model for WHO’s system is the European Antimicrobial Resistance Surveillance Network (EARS-Net). When it was conceived, it faced big challenges, ranging from scepticism that laboratory data were comparable across countries to doubts about the usefulness of comparing data from countries with vastly different healthcare systems and diagnostic practices.^4^ In the end, EARS-Net has robust participation across all European Union member states and has become a strong advocacy tool for antibiotic policy. The idea of EARS-Net was inspired by a ready supply of relatively reliable results from antibiotic susceptibility tests that were conducted routinely in the course of patient care. Using the data for surveillance added value to their original purpose - to guide patient treatment - without incurring additional laboratory costs. Collection and analysis is not free, but the cost is small in comparison to the sunk laboratory costs.

These aggregated laboratory data - expressed as the proportion of isolates resistant to specific antibiotics - are useful for broad policy purposes, particularly when paired with antibiotic consumption data which can be collected in Europe. Together, these complementary surveillance inputs provide ecological level evidence that antibiotic consumption is causally related to AMR prevalence.^5^ However, even together, these information streams do not support the development of targeted AMR transmission and infection control strategies.

Current concepts such as the OneHealth approach embody the idea of an increasingly connected world and emphasise the importance of controlling antibiotic exposure in all microbial habitats - humans, animals and the environment. It has become clear that the dissemination of AMR is strongly associated with the expansion of highly adapted bacterial lineages (‘high-risk clones’) or successful and transferrable genetic elements. It is therefore crucial for surveillance systems to capture the transmission dynamics at more informative epidemiological scales as well as across ecological interfaces.

Crucial to understanding transmission dynamics driven by ecological constraints, for example drug pressure, migration, trade, climate change, etc. are sampling strategies that would provide snapshots of microbial populations at different points in time. WGS of bacterial pathogens has already proven useful for epidemiological surveillance, outbreak detection and infection control.^6,7,8,9^ As its use becomes more widespread, the need for standard sampling techniques, standard operating procedures and standardised analysis will increase in importance. More well-trained personnel both in the laboratory and bioinformatics aspects will be needed as well as, online tools and software that will facilitate the routine analysis of data.^10^

Where does that lead us in pushing forward with AMR surveillance in Africa in 2018? Regarding what we are considering ‘conventional’ laboratory-based surveillance, an important question is whether the laboratory results being produced routinely would, if aggregated, provide useful policy guidance. A 2016 evaluation of the World Bank-supported East African Public Health Laboratory Networking project, which includes about 30 clinical laboratories in five countries (Kenya, Tanzania, Uganda, Rwanda, and Burundi), demonstrated shortfalls in adequately trained personnel, as well as stockouts of key material resources.^11^ These lead to shortcomings in the utilisation of diagnostic services caused by long turn-around times and a perceived lack of clinical relevance for therapeutic choice. Clearly, under these circumstances, current investigation habits will lead to an overestimation of resistance and will not be able to provide the data needed to inform local or national AMR containment strategies.

## Why whole genome sequencing surveillance?

We argue that WGS could provide a level of scrutiny elusive to conventional AMR surveillance systems. What WGS offers, is the ability to appraise the evolutionary blueprints that reveal the genetic composition of currently extant pathogens. Tremendous insights into multi-drug resistant typhoid - one of Africa’s most important pathogens - have already been gained through WGS, and the need to continue tracking typhoid infection patterns will not diminish any time soon.^10^ Thus, WGS data would provide an insight into the genomic population structure and pinpoint clones of public health importance irrespective of the representativeness of the original sample. High-risk clones can be readily identified by their clonal relatedness, abundance, geographic clustering as well as by their genetic contents, that is, virulence genes, antibiotic resistance genes and the like. In this way, even biased samples would allow the discovery of emerging lineages, transmission and spread, and would provide invaluable benefits for understanding the origin and management of epidemics. Whole genome sequencing generates biologically meaningful, robust and portable data. Their relevance is increasing over time as national and international datasets grow and provide historical, evolutionary, ecological and epidemiological contexts with increasing granularity. It is therefore high time for African health systems to consider the necessary steps toward the collection and analysis of samples from patients, livestock and wild animals, and the environment for analysis by WGS.

The expertise and some facilities for WGS already exist in several countries in sub-Saharan Africa, and we would propose that the enabled laboratories themselves devise a plan for a pilot programme. This could be kicked off by a foundation-funded brainstorming meeting bringing together Africa’s leading genome scientists who represent the institutions with existing capacity. The initial project could be in one country with sampling in different areas over the course of a year, or something more ambitious. The vision should encompass all infectious agents with public health relevance including susceptible bacteria, viruses and parasites threatening the health of humans and animals. The programme would require external funding and possibly collaboration from scientists engaged in similar activities in Europe or elsewhere. In addition to strengthening the science and producing information of continent-wide relevance, a coordinated continent-wide effort will be better able than small efforts to avoid certain pitfalls, have greater bargaining power with equipment and supply companies, and enjoy sharing of scarce human resources, for example bioinformaticians. It is particularly important, given the periodic improvements in technology (and the continuous declines in cost), that a sustainable plan for equipment updating be in place. It is, in 2018, however, feasible and reasonable to begin this effort and that it be Africa led.

### Conclusion

Surveillance using WGS can provide direct insight into the evolution and spread of antimicrobial resistant pathogens and complements traditional laboratory-based surveillance. The scientific capacity and equipment are already present in several African countries to conduct WGS-based surveillance, and there is no reason to delay utilising it and every reason to launch the effort without delay.
